# Anteroposterior axis of the tibia for kinematic aligned total knee arthroplasty

**DOI:** 10.1002/jeo2.70087

**Published:** 2024-11-22

**Authors:** Seikai Toyooka, Noriaki Arai, Hironari Masuda, Hirotaka Kawano, Takumi Nakagawa

**Affiliations:** ^1^ Department of Orthopaedic Surgery Teikyo University School of Medicine Tokyo Japan

**Keywords:** Akagi line, kinematic alignment, posterior condylar axis, tibia rotation, total knee arthroplasty

## Abstract

**Purpose:**

It is not known where the anatomical axis of rotation on the tibial side will be in kinematic alignment (KA), a rapidly expanding area of total knee arthroplasty (TKA) alignment technique today. The purpose of this study was to define the tibial axis for KA‐TKA.

**Methods:**

Fifty patients who underwent computed tomography (CT) examination of the lower extremities at a single institution were included. The posterior condylar axis (PCA) and surgical epicondylar axis (SEA) were identified in the CT axial view and projected onto the tibial slice. The respective perpendicular lines that pass through was attachment of the posterior cruciate ligament (PCL) were identified as the anatomic axis of rotation of the tibia relative to the PCA and SEA, and the position of each axis of rotation. Furthermore, the relationship of these perpendicular lines with the Akagi line was evaluated.

**Results:**

The axis of tibial rotation to the SEA was similar to that of the Akagi line; the axis of tibial rotation to the PCA was located approximately 2.9° medial to the Akagi line, and when the origin of the tibial axis was set at the PCL attachment site, the intersection point of the tibial axis was approximately 2.5 mm medial to the medial border of the tibial tuberosity. The distribution of tibial axis had a wide range.

**Conclusion:**

Although there is a large individual variation, the average tibial axis for KA‐TKA is 2.9° more internally rotated than the Akagi line.

**Level of Evidence:**

Level IV.

## Graphical abstract

The anatomical tibial axis in kinematic alignment (KA) total knee arthroplasty was investigated from computed tomography images. Although there is a great deal of individual variation, the average tibial axis in KA is 2.9° more internally rotated than the Akagi line passing through the medial border of the patella tendon from the posterior cruciate ligament.

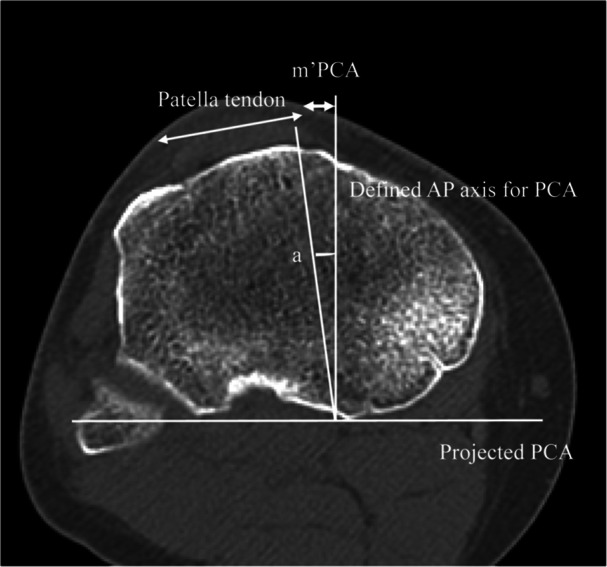

**Abbreviations**
KA: kinematic alignment
MA: mechanical alignment
PCA: posterior condylar axis
PCL: posterior cruciate ligament
SEA: surgical epicondylar axis
TKA: total knee arthroplasty

## INTRODUCTION

The rotational relationship between the femoral and tibial components is an important factor affecting the overall function and durability of total knee arthroplasty (TKA). Malrotation of tibial implants can lead to aseptic loosening, instability, polyethylene wear, and extension deficits, resulting in revision TKA [1, 10, 19, 23, 24]. It is not difficult to imagine that a mismatch with femoral rotation reduces implant conformity, causing patient discomfort and implant dysfunction and reducing patient satisfaction [19, 24].

To date, the mechanical alignment (MA) technique with osteotomy perpendicular to the mechanical axis has been the gold standard for TKA. In this technique, the most commonly used centre of rotation on the femoral side is the surgical epicondylar axis (SEA), which connects the sulcus of the medial femoral epicondyle and the lateral femoral epicondyle [5, 8, 13, 16, 17]. The most appropriate axis for the tibial side in relation to this femoral axis is the Akagi line, which connects the attachment site of the posterior cruciate ligament (PCL) to the medial border of the tibial tuberosity and is used by many surgeons as an indicator [3, 4]. On the other hand, kinematic alignment (KA), which attempts to rebuild the patient's constitutional alignment, has become widespread. In this technique, the centre of rotation on the femoral side is set on the cylindrical axis [11]. Specifically, this is done by matching the amount of osteotomy on the femoral posterior condyle both medially and laterally, referring to the posterior condylar axis (PCA). At present, although the axis of rotation on the femoral side has been determined, it is not known where the axis on the tibial side will be in KA.

The purpose of this study was to define the anatomical tibial axis for KA of the TKA. Identifying this may allow for tibial implants that match the tibial axis corresponding to the femoral axis created based on cylindrical axes in KA. Previous anatomical studies have reported that the PCA is internally rotated relative to the SEA. Therefore, we hypothesise that the AP axis of the tibia in KA‐TKA involves slight internal rotation compared with the Akagi line.

## MATERIALS AND METHODS

### Patients and design

The study protocol was approved by the institutional review board of the Teikyo University Ethics Committee, and all patients provided informed consent.

Fifty patients who had computed tomography (CT) scans of the lower extremities, including the knee joint, at a single institute between January 2023 and December 2023 were included. Patients whose bone geometry was affected by the following conditions were excluded: under 20 years of age, a history of lower extremity fracture, a history of lower extremity surgery, a history of patellar dislocation or subluxation, bone tumour, knee osteoarthritis according to the Kellgren and Lawrence classification greater than 2°, osteonecrosis, or inflammatory disease such as rheumatoid arthritis.

The patients were informed of the risk of radiographic exposure during CT scans, and written informed consent was obtained.

### Imaging protocol

CT (Toshiba Aquilion; Toshiba Medical Systems Corporation) was performed using the standard bone CT protocol with 0.5‐mm axial sections in three planes, with a tube voltage of 120 kV. During scanning, the leg was set fixed in a wooden frame so that the knee was kept in an extended position (0° of flexion) and placed parallel to the CT bed. The knee position was determined so that the subject could feel a naturally extended knee position in the frame without any feeling of internal or external rotation. Scans were made perpendicular to the tibial shaft axis at 3 mm intervals with a 2‐mm wide source beam.

### Radiographic analysis

**Figure 1:**
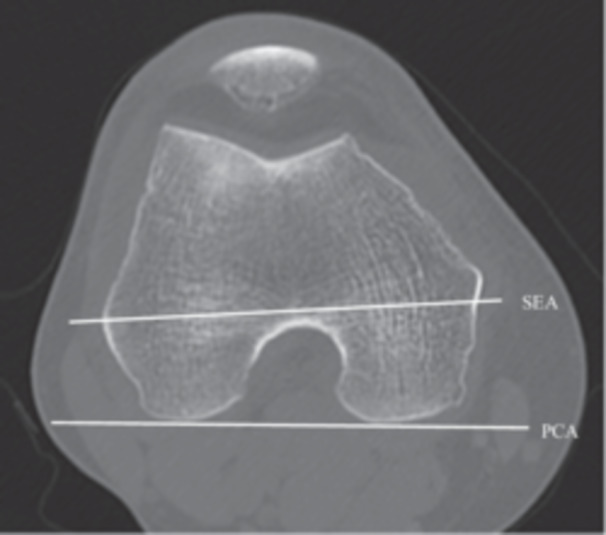
In the coronal slice of the CT image, the slice containing the lowermost point of the femoral posterior condyle was selected, and the line connecting the lowermost points of the medial and lateral posterior condyles was defined as the PCA. In addition, a slice was selected in the vicinity of the slice where the grooves of the lateral femoral epicondyle and medial femoral epicondyle could be seen, and the line connecting them was defined as the SEA. CT, computed tomography; PCA, posterior condylar axis; SEA, surgical epicondylar axis.

The CT images taken were analysed to identify the axis of the tibia relative to the PCA and SEA. The evaluation was conducted using a PACS workstation (V5 Impax, Agfa HealthCare). These were used for overlaying CT scans spatially, for drawing lines or points on CT scans, for projecting a line or a point on a scan to another scan, for measuring relative widths, and for measuring the angles between two lines. The following measurements were made with reference to the method previously used to identify the Akagi line [4]. First, the slice containing the lowest point of the femoral posterior condyle was selected in the axial slice of the femur, and the line connecting the lowest point of the medial and lateral posterior condyles was used as the PCA. In the region of that axial slice, a slice in which the lateral epicondyle and the sulcus of the medial femoral epicondyle could be identified was selected, and the line connecting them was used as the SEA (Figure 1).

**Figure 2:**
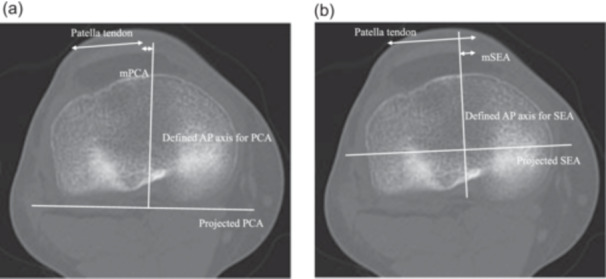
PCA and SEA were projected onto coronal slices at the level of the tibial articular plane. A line perpendicular to the projected PCA and passing through the centre of the PCL attachment was defined as the AP axis of the tibial PCA. Similarly, a line perpendicular to the projected SEA and passing through the centre of the PCL attachment was defined as the AP axis of the SEA of the tibia. mPCA (mm) was defined as the distance from the tibial axis of the PCA to the medial edge of the patellar tendon, and mSEA (mm) as the distance from the tibial axis of the SEA to the medial edge of the patellar tendon. (a) Measurement of the mPCA. In a slice of the tibial articular surface, the line perpendicular to the projected PCA that passes through the attachment of the PCL was defined as the AP axis for PCA, and the distance from the medial border of the patella tendon was defined as mPCA. (b) Measurement of the mSEA. In the same slice, a line perpendicular to the projected SEA that passes through the attachment of the PCL was defined as the AP axis for SEA, and the distance from the medial border of the patella tendon was defined as m'SEA. AP, anterior–posterior; PCA, posterior condylar axis; PCL, posterior cruciate ligament; SEA, surgical epicondylar axis.

First, to identify the tibial axis at the level of the tibial articular surface, the PCA and SEA were projected onto the axial slice of the tibial articular surface. A line perpendicular to the projected PCA and passing through the centre of the PCL attachment was defined as the AP axis for the PCA of the tibia. Similarly, a line perpendicular to the projected SEA and passing through the centre of the PCL attachment was defined as the AP axis for the SEA of the tibia. The PCL was clearly observed in the posterior condylar notch of the tibia. To measure the distance from the medial border of the patellar tendon to the tibial axis at the level of the tibial articular surface, the distance between the tibial axis of the PCA and the medial border of the patellar tendon was defined as the mPCA (mm), and the distance between the tibial axis of the SEA and the medial border of the patellar tendon was defined as the mSEA (mm) (Figure 2). The intersection of the tibial axis and the patellar tendon was defined as negative when it was on the patellar tendon side and positive when it was medial to the patellar tendon.

**Figure 3:**
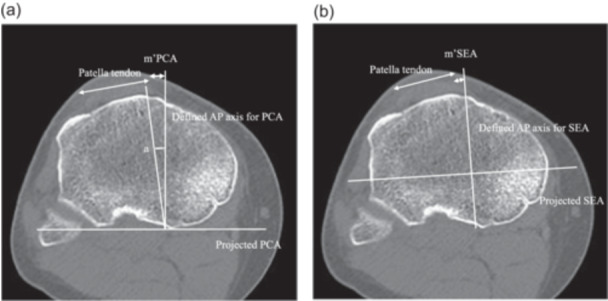
The PCA and SEA were projected onto the axial slice where the patellar tendon attaches to the tibial tuberosity. At this level, the distance between the tibial axis and the medial edge of the patellar tendon relative to the PCA was defined as m'PCA (mm) and the distance between the tibial axis and the medial edge of the patellar tendon relative to the SEA was defined as m'SEA (mm). (a) Measurement of the m'PCA and angle A. In the slice where the patella tendon attaches to the tibial tuberosity, the AP axis for PCA was defined as a line that is perpendicular to the projected PCA and passes through the PCL, connected to the medial border of the patella tendon. The distance was defined as m'PCA. In addition, the angle between Akagi line and AP axis for PCA was defined as angle A. (b) Measurement of the m'SEA. In the same slice, the line perpendicular to the projected SEA that passes through the attachment of the PCL was defined as the AP axis for SEA, and the distance to the medial border of the patella tendon was defined as mSEA. AP, anterior–posterior; PCA, posterior condylar axis; PCL, posterior cruciate ligament; SEA, surgical epicondylar axis.

Second, to identify the tibial axis at the level where the patellar tendon attaches to the tibial tuberosity, the PCA and SEA were projected onto the axial slice where the patellar tendon attaches to the tibial tuberosity. The mPCA (mm) of the slice at the level of the tibial tuberosity was defined as m'PCA, and the mSEA of the slice at the level of the tibial tuberosity was defined as m'SEA (mm) (Figure 3). Finally, to determine the angle between the tibial AP axis of the PCA and the Akagi line, the angle between the line connecting the centre of the PCL to the medial border of the patellar tendon and the tibial axis of the PCA was measured at the level of the tibial attachment and defined as angle a (degrees).

Measurements of the mPCA, mSEA, m'PCA, and m'SEA were performed by one observer (ST). Intraobserver variations in the measurements were assessed by repeating the measurements 10 times in three subjects. The maximum intraobserver difference in the measurements was 5%, and the largest standard deviation was 2.2%. The angle between the AP axis and the Akagi line was measured by three independent observers (ST, NA and HM). All angular measurements were repeated by the three observers, and the mean was regarded as the true value. The maximum interobserver difference was less than 2.0°.

## RESULTS

Fifty knees from 48 patients were enroled in this study. Patient demographics are shown in Table 1. The mean patient age was 52.0 (20–77) years. Twenty‐seven knees were examined via CT after minor trauma, 17 knees were evaluated closely for knee pain, and 6 knees were evaluated for soft tissue masses around the knee joint.

**Table 1:** Patient demographics.

*n*	50 knees (48 patients)
Age	Mean, 52.0 (20–77)
Height (cm)	Mean, 164.8 (148–88)
Weight (kg)	Mean, 71.0 (39–148)
BMI	Mean, 25.6 (17.0–41.8)
Sex	Male: 26, female: 22
Reason of CT	Minor trauma: 27 Scrutiny of pain: 17 Soft tissue tumour: 6

Abbreviations: BMI, body mass index; CT, computed tomography.

At the level of the tibial plateau, the AP axis of the PCA generally passed through the proximity of medial border of the patellar tendon, averaging 1.0 ± 2.6 mm medial to the medial border of the patellar tendon. In men, it was 0.6 ± 2.5 mm medial to the medial border of the patellar tendon, and in women, it was 1.4 ± 3.0 mm medial to the medial border of the patellar tendon. At this level, the AP axis of the defined SEA generally passed above the patellar tendon itself and was on average −1.2 ± 2.8 mm lateral to the medial border of the patellar tendon. In men, it was −1.5 ± 2.8 mm lateral to the medial border of the patellar tendon, and in women, it was −0.8 ± 2.8 mm lateral to the medial border of the patellar tendon.

**Figure 4:**
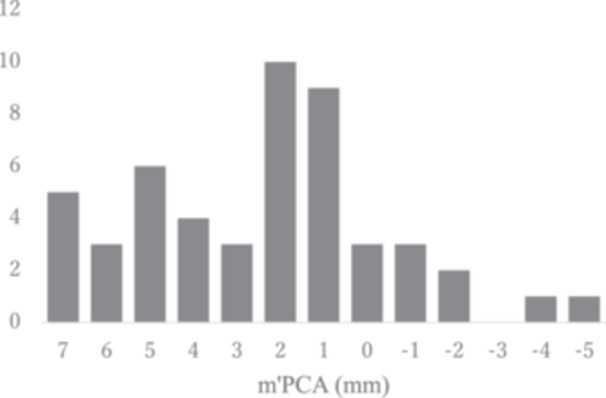
Distribution of m'PCA. Vertical axis is the number of *n*, horizontal axis is the distance of m'PCA (mm). PCA, posterior condylar axis.

At the level of the patellar tendon attachment, the tibial AP axis of the PCA passed an average of 2.5 ± 3.0 mm medial to the medial border of the tibial tuberosity. The distribution of the results is shown in Figure 4. In men, it was 2.3 ± 3.0 mm medial to the medial edge of the patellar tendon, and in women, it was 2.7 ± 3.1 mm medial. At this level, the defined AP axis of the SEA generally passed near the medial border of the patellar tendon and was on average 0.7 ± 2.9 mm medial to the medial border of the tibial tuberosity. In men, it was 0.4 ± 2.9 mm medial to the medial border of the tibial tuberosity, and in women, it was 1.0 ± 2.8 mm medial to the medial border of the tibial tuberosity. In every case, the AP axis of the tibia passed more medially in women than in men.

**Figure 5:**
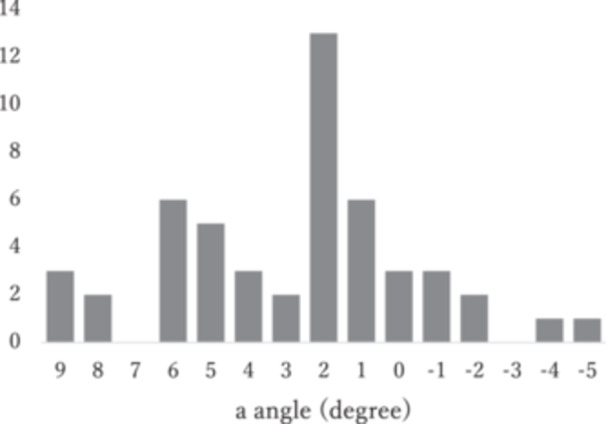
Distribution of the angle between the tibial AP axis and the Akagi line relative to the PCA. Vertical axis is the number of *n*, horizontal axis is the angle (°). AP, anterior–posterior; PCA, posterior condylar axis.

The mean angle which is between the AP axis for PCA and Akagi line was 2.9 ± 3.3° to the medial edge of the patellar tendon in all subjects. The distribution is shown in Figure 5. The mean angle was 2.5 ± 3.2° for males and 3.4 ± 3.5° for females.

## DISCUSSION

The most important finding of this study was that when the femoral axis of rotation was set at the PCA, the tibial axis at the level of the patellar tendon attachment was approximately 2.9° internally rotated to the Akagi line, and when the origin of the tibial axis was set at the PCL attachment site, the intersection of the tibial axis was approximately 2.5 mm medial to the medial border of the tibial tuberosity. Akagi et al. showed that when the PCL attachment site, which can be easily identified on CT, is used as a landmark, the medial border of the patellar attachment site is an indicator of the tibial axis [3, 4]. In the present study, we measured the tibial axis to the SEA using the same method and found that it was 0.7 mm medial to the medial border of the patellar tendon attachment site, almost the same as that reported by Akagi et al. When the tibial axis to the PCA was measured in the present study, it was medialized by an average of 2.9° compared to the Akagi line. Previous studies have reported that the PCA is internally rotated 1–4° relative to the SEA [2, 5, 9, 12, 18, 20, 26, 28]. Considering this, the results of the present study are reasonable.

Conventionally, the reference axis of rotation on the femoral side was the SEA, and the corresponding reference axis on the tibial side was the Akagi line. However, in the currently widely practised KA method, the reference axis on the femoral side is the PCA. Currently, many surgeons still use the Akagi line or 1/3 tibial position as the reference axis for tibial rotation when performing KA [3, 6, 21]. The results of this study show that when the Akagi line is used for KA, the average femoral‐tibial mismatch is 2.9°, and the mismatch is much greater when the point 1/3 of the tibial tuberosity is used as the reference for KA. With less restrictive implants, rotational mismatch could lead to subluxation of the tibiofemoral joint and wear of the polyethylene insert. With more restrictive implants, impingement of the implant and insert can occur, leading to misalignment of the knee joint, ankle and foot. Conversely, excessive internal rotation of the tibial component is a risk factor for pain and worse outcomes [27]. For this reason, care should be taken to avoid unwanted internal rotation. Clinical data on this topic is not yet available but should be discussed further as KA expands.

The second important finding is the wide range of tibial axis values relative to the PCA. As shown in Figures 4 and 5, the tibial axis can be 5° more externally rotated or 9° more internally rotated than the Akagi line. Individual differences in the morphology of the lateral femoral condyles have been reported to be significant, which may be one of the reasons for the large variation in the present results [14, 25]. The present results provide one criterion for the tibial rotation axis in KA‐TKA, but it is not an index that applies to all individuals. In some cases, tibial rotation requires not only anatomical but also kinematic techniques, such as checking the conformity of the tibial component to the femoral component during repeated flexion‐extension of the knee.

At the level of the articular surface of the tibia, the tibial axis for the SEA passed lateral to the medial border of the patella, whereas the tibial axis for the PCA passed near the medial border of the patella. Previously, Akagi et al. reported that at the level of the articular surface, the tibial axis of rotation for SEA passes 11% lateral to the width of the patellar tendon rather than the medial border of the patellar tendon, which is similar to the present results [4]. In the future, if implants are able to fully reproduce the original tibial shape via KA, the axis of rotation of the tibia will pass through the medial border of the patellar tendon. Although it is still difficult to completely recreate the original knee with current technology, the present results may provide guidance for future advances in robotics and implants, as well as for the prediction of original knee morphology using the CPAK classification and other methods [15].

The present study had several limitations. The number of subjects was limited (the total number of subjects was 50 knees, 27 males and 23 females). Additionally, the study was limited to Japanese subjects. The data included in this study may be typical for Asian knees, and there may be anatomic differences in the Caucasian population. Therefore, the results of this study should be interpreted with caution. Other limitations are due to the method of measurement. The knee has a screw home mechanism that rotates the lower leg externally during extension. In this study, images were taken in the knee joint extension position, where rotation of the femur and tibia itself is unlikely to occur. In addition, to minimise the effects of rotation, images were taken in what the examiner determined to be in a natural extended position. However, even in the extended position, a certain degree of rotational misalignment may have occurred. Although the image evaluation in this study verified both intraobserver and interobserver errors, it is possible that this rotation misalignment may have affected the results. This study was based on image metrics, and it remains to be seen whether these metrics can be adapted to actual patient knees. Finally, one of the main problems with tibial internal rotation in TKA is the high risk of patellofemoral dislocation, which was not evaluated in this study [7, 22]. Future studies using 3D or in‐motion images would be desirable.

## CONCLUSION

Although there is a large individual variation, the average tibial axis for KA‐TKA is 2.9° more internally rotated than that of the Akagi line.

## AUTHOR CONTRIBUTIONS

*Conceptualisation*: Hirotaka Kawano and Takumi Nakagawa. *Data analysis and interpretation*: Noriaki Arai and Hironari Masuda. *Writing—original draft*: Seikai Toyooka. All authors critically reviewed and revised the manuscript draft and approved the final version for submission.

## CONFLICT OF INTEREST STATEMENT

The authors declare no conflict of interest.

## ETHICS STATEMENT

This study was performed in accordance with the principles of the Declaration of Helsinki. The Ethics Committee of Teikyo University granted approval (approval number: 19‐174‐2). Written informed consent was obtained from all patients.

## Data Availability

The authors will make data and materials supporting the results or analyses presented in the paper available upon reasonable request.

## References

[1] Abdelnasser, M.K. , Adi, M.M. , Elnaggar, A.A. & Tarabichi, S. (2020) Internal rotation of the tibial component in total knee arthroplasty can lead to extension deficit. Knee Surgery, Sports Traumatology, Arthroscopy, 28(9), 2948–2952. Available from: 10.1007/s00167-019-05695-w 31482183

[2] Aglietti, P. , Sensi, L. , Cuomo, P. & Ciardullo, A. (2008) Rotational position of femoral and tibial components in TKA using the femoral transepicondylar axis. Clinical Orthopaedics and Related Research, 466, 2751–2755. Available from: 10.1007/s11999-008-0452-8 18825470 PMC2565051

[3] Akagi, M. , Mori, S. , Nishimura, S. , Nishimura, A. , Asano, T. & Hamanishi, C. (2005) Variability of extraarticular tibial rotation references for total knee arthroplasty. Clinical Orthopaedics and Related Research, 436, 172–176. Available from: 10.1097/01.blo.0000160027.52481.32 15995437

[4] Akagi, M. , Oh, M. , Nonaka, T. , Tsujimoto, H. , Asano, T. & Hamanishi, C. (2004) An anteroposterior axis of the tibia for total knee arthroplasty. Clinical Orthopaedics and Related Research, 420, 213–219. Available from: 10.1097/00003086-200403000-00030 15057100

[5] Asano, T. , Akagi, M. & Nakamura, T. (2005) The functional flexion‐extension axis of the knee corresponds to the surgical epicondylar axis. The Journal of Arthroplasty, 20(8), 1060–1067. Available from: 10.1016/j.arth.2004.08.005 16376264

[6] Berger, R.A. & Crossett, L.S. (1998) Determining the rotation of the femoral and tibial components in total knee arthroplasty: a computer tomography technique. Operative Techniques in Orthopaedics, 8(3), 128–133. Available from: 10.1016/S1048-6666(98)80022-0

[7] Bonnin, M. , Saffarini, M. , Lustig, S. & Hirschmann, M.T. (2024) Decoupling the trochlea from the condyles in total knee arthroplasty: the end of a curse? Knee Surgery, Sports Traumatology, Arthroscopy, 32(7), 1645–1649. Available from: 10.1002/ksa.12267 38769816

[8] Churchill, D.L. , Incavo, S.J. , Johnson, C.C. & Beynnon, B.D. (1998) The transepicondylar axis approximates the optimal flexion axis of the knee. Clinical Orthopaedics and Related Research, 356, 111–118. Available from: 10.1097/00003086-199811000-00016 9917674

[9] Conti, M.S. , Kleeblad, L.J. , Jones, C.W. , Pearle, A.D. & Sculco, P.K. (2019) Distal femoral rotation is not associated with preoperative proximal tibial varus angle in patients with isolated medial compartment osteoarthritis. The Journal of Arthroplasty, 34(2), 281–285. Available from: 10.1016/j.arth.2018.09.080 30377013

[10] De Valk, E.J. , Noorduyn, J.C.A. & Mutsaerts, E.L.A.R. (2016) How to assess femoral and tibial component rotation after total knee arthroplasty with computed tomography: a systematic review. Knee Surgery, Sports Traumatology, Arthroscopy, 24(11), 3517–3528. Available from: 10.1007/s00167-016-4325-5 27655141

[11] Howell, S.M. , Akhtar, M. , Nedopil, A.J. & Hull, M.L. (2024) Reoperation, implant survival, and clinical outcome after kinematically aligned total knee arthroplasty: a concise clinical follow‐up at 16 years. The Journal of Arthroplasty, 39(3), 695–700. Available from: 10.1016/j.arth.2023.08.080 37659680

[12] Jabalameli, M. , Moradi, A. , Bagherifard, A. , Radi, M. & Mokhtari, T. (2016) Evaluation of distal femoral rotational alignment with spiral CT scan before total knee arthroplasty (a study in Iranian population). The Archives of Bone and Joint Surgery, 4(2), 122–127. 27200388 PMC4852036

[13] Jang, E.S. , Davignon, R. , Geller, J.A. , Cooper, H.J. & Shah, R.P. (2023) Reference axes for tibial component rotation in total knee arthroplasty: computed tomography‐based study of 1,351 tibiae. Journal of Bone and Joint Surgery, 105(1), 1–8. Available from: 10.2106/JBJS.22.00520 36367766

[14] Li, K. , Langdale, E. , Tashman, S. , Harner, C. & Zhang, X. (2012) Gender and condylar differences in distal femur morphometry clarified by automated computer analyses. Journal of Orthopaedic Research, 30, 686–692. Available from: 10.1002/jor.21575 22025249 PMC3290733

[15] MacDessi, S.J. , Griffiths‐Jones, W. , Harris, I.A. , Bellemans, J. & Chen, D.B. (2021) Coronal plane alignment of the knee (CPAK) classification. The Bone & Joint Journal, 103–b(2), 329–337. Available from: 10.1302/0301-620X.103B2.BJJ-2020-1050.R1 PMC7954147 33517740

[16] Olcott, C.W. & Scott, R.D. (1999) Femoral component rotation during total knee arthroplasty. Clinical Orthopaedics & Related Research, 367, 39–42. 10546596

[17] Oussedik, S. , Scholes, C. , Ferguson, D. , Roe, J. & Parker, D. (2012) Is femoral component rotation in a TKA reliably guided by the functional flexion axis? Clinical Orthopaedics and Related Research, 470, 3227–3232. Available from: 10.1007/s11999-012-2515-0 22895689 PMC3462881

[18] Pagnano, M.W. & Hanssen, A.D. (2001) Varus tibial joint line obliquity: a potential cause of femoral component malrotation. Clinical Orthopaedics and Related Research, 392, 68–74. 11716427

[19] Panni, A.S. , Ascione, F. , Rossini, M. , Braile, A. , Corona, K. , Vasso, M. et al. (2018) Tibial internal rotation negatively affects clinical outcomes in total knee arthroplasty: a systematic review. Knee Surgery, Sports Traumatology, Arthroscopy, 26(6), 1636–1644. Available from: 10.1007/s00167-017-4823-0 29247357

[20] Park, A. , Nam, D. , Friedman, M.V. , Duncan, S.T. , Hillen, T.J. & Barrack, R.L. (2015) Inter‐observer precision and physiologic variability of MRI landmarks used to determine rotational alignment in conventional and patient‐specific TKA. The Journal of Arthroplasty, 30(2), 290–295. Available from: 10.1016/j.arth.2014.08.015 25267537 PMC4323956

[21] Roper, G.E. , Bloemke, A.D. , Roberts, C.C. , Spangehl, M.J. & Clarke, H.D. (2013) Analysis of tibial component rotation following total knee arthroplasty using 3D high definition computed tomography. The Journal of Arthroplasty, 28(8), 106–111. Available from: 10.1016/j.arth.2013.04.053 23906867

[22] Shatrov, J. , Coulin, B. , Batailler, C. , Servien, E. , Walter, B. & Lustig, S. (2023) Alignment philosophy influences trochlea recreation in total knee arthroplasty: a comparative study using image‐based robotic technology. International Orthopaedics, 47(2), 329–341. Available from: 10.1007/s00264-022-05570-3 36112197 PMC9877070

[23] Skowronek, P. , Arnold, M. , Starke, C. , Bartyzel, A. , Moser, L.B. & Hirschmann, M.T. (2021) Intra‐ and postoperative assessment of femoral component rotation in total knee arthroplasty: an EKA knee expert group clinical review. Knee Surgery, Sports Traumatology, Arthroscopy, 29(3), 772–782. Available from: 10.1007/s00167-020-06006-4 32350578

[24] Steinbrück, A. , Schröder, C. , Woiczinski, M. , Müller, T. , Müller, P.E. , Jansson, V. et al. (2016) Influence of tibial rotation in total knee arthroplasty on knee kinematics and retropatellar pressure: an in vitro study. Knee Surgery, Sports Traumatology, Arthroscopy, 24, 2395–2401. Available from: 10.1007/s00167-015-3503-1 25577221

[25] Terzidis, I. , Totlis, T. , Papathanasiou, E. , Sideridis, A. , Vlasis, K. & Natsis, K. (2012) Gender and side‐to‐side differences of femoral condyles morphology: osteometric data from 360 caucasian dried femori. Anatomy Research International, 2012, 1–6. Available from: 10.1155/2012/679658 PMC3437276 22970374

[26] Thienpont, E. , Schwab, P.E. , Paternostre, F. & Koch, P. (2014) Rotational alignment of the distal femur: anthropometric measurements with CT‐based patient‐specific instruments planning show high variability of the posterior condylar angle. Knee Surgery, Sports Traumatology, Arthroscopy, 22(12), 2995–3002. Available from: 10.1007/s00167-014-3086-2 24888223

[27] Valkering, K.P. , Breugem, S.J. , Van den Bekerom, M.P. , Tuinebreijer, W.E. & Van Geenen, R.C.I. (2015) Effect of rotational alignment on outcome of total knee arthroplasty: a systematic review of the literature and correlation analysis. Acta Orthopaedica, 86(4), 432–439. Available from: 10.3109/17453674.2015.1022438 25708694 PMC4513597

[28] Victor, J. (2009) Rotational alignment of the distal femur: a literature review. Orthopaedics & Traumatology: Surgery & Research, 95(5), 365–372. Available from: 10.1016/j.otsr.2009.04.011 19592323

